# Supplementary material for: Association between cardiometabolic risk factors and multidrug-resistant tuberculosis: A case-control study
Source: PLoS One. 2025 Sep 26;20(9):e0333362. doi: 10.1371/journal.pone.0333362 (PMC12469359; doi:10.1371/journal.pone.0333362)
Supplement: Attachment — Submitted filename: Response To Reviewers.docx [file pone.0333362.s001.docx]

**Response to Reviewers**

**Manuscript Title**: Cardiometabolic risk factors associated with multidrug resistant tuberculosis: A case-control study
**Manuscript ID**: PONE-D-25-24268

Dear Editors,

Thank you for your feedback on our manuscript. Please find below our point-by-point response to the editorial comments:

**Comment:**
Based on our evaluation of your manuscript and the information you provided, we do not feel that your submission meets our ethical requirements for human subjects research submissions. PLOS ONE requires that research meets all applicable standards for the ethics of experimentation and research integrity (http://journals.plos.org/plosone/s/human-subjects-research). We reserve the right to reject any submission that does not meet our internal ethical standards, which in some cases are more stringent than local ethical standards.

**Response:**
We sincerely apologize for the confusion caused by the wording in the original manuscript. The date mentioned (1 September) referred to the final submission of protocol, not participant recruitment. Ethical approval from the Nepal Health Research Council (NHRC) was **obtained before** any human participants were enrolled or data were collected.

To address this, we have revised the **Methods section** to clarify the study timeline and ensure there is no ambiguity regarding the timing of ethical approval and recruitment.

We appreciate the opportunity to clarify this point and reaffirm our commitment to upholding the highest ethical standards.

Sincerely,
Dr. Sishir Poudel
MBBS, BPKIHS
2025-05-29
